# Realism, reliability, and epistemic possibility: on modally interpreting the Benacerraf–Field challenge

**DOI:** 10.1007/s11229-020-02984-7

**Published:** 2020-12-21

**Authors:** Brett Topey

**Affiliations:** Philosophy Department (KGW), University of Salzburg, Salzburg, Austria

**Keywords:** Reliability challenge, Platonism, Modal epistemology, Counterpossibles

## Abstract

A Benacerraf–Field challenge is an argument intended to show that common realist theories of a given domain are untenable: such theories make it impossible to explain how we’ve arrived at the truth in that domain, and insofar as a theory makes our reliability in a domain inexplicable, we must either reject that theory or give up the relevant beliefs. But there’s no consensus about what would count here as a satisfactory explanation of our reliability. It’s sometimes suggested that giving such an explanation would involve showing that our beliefs meet some modal condition, but realists have claimed that this sort of modal interpretation of the challenge deprives it of any force: since the facts in question are metaphysically necessary and so obtain in all possible worlds, it’s trivially easy, even given realism, to show that our beliefs have the relevant modal features. Here I show that this claim is mistaken—what motivates a modal interpretation of the challenge in the first place also motivates an understanding of the relevant features in terms of epistemic possibilities rather than metaphysical possibilities, and there are indeed epistemically possible worlds where the facts in question don’t obtain.

## Introduction

For us to be correct in our beliefs about some part of the world is for it to be the case that, in the relevant domain, our beliefs by and large coincide with the facts. So we might wonder: what explains this coincidence?

Depending on what domain is in question, an answer may be easy to come by. For instance, a straightforward causal explanation is available in the case of my beliefs about the weather: I came to my belief that it’s raining by observing the rain. But connections of the right sort between our beliefs and the facts aren’t always so easy to make out. This isn’t always a reason for concern—we may be unable to find a connection simply because we’re not aware of our beliefs’ full causal histories. For example, I don’t recall how I came to have the beliefs I do about the atmosphere of the planet Venus and so can’t provide a causal explanation of the coincidence between those beliefs and the facts, but I’m nevertheless quite confident that some such explanation is available. In some cases, though, the difficulty runs deeper: sometimes it seems that we’re unable to provide an explanation not because we lack information but because there just isn’t an explanation to be had—our overall theory of the world and our place in it seems to entail that there’s no connection, causal or otherwise, between the relevant part of the world being what it is and our beliefs being what they are. And such cases do seem worrisome.

For instance, one standard view about mathematical facts is a kind of platonism: mathematical facts are facts about a realm of mind-independent abstract objects. But there can’t, on any naturalistically respectable theory of how our cognitive faculties work, be a connection of any sort between mind-independent abstract objects and our having the mathematical beliefs we do. So it seems that, given platonism, no explanation is going to be available, even in principle, of why our mathematical beliefs coincide with the facts. And this suggests that, according to our own theory of the world and our place in it, any coincidence between our mathematical beliefs and the mathematical facts is completely accidental.

One response to this information would be simply to celebrate our extraordinary good fortune, to insist that, in the face of incredibly long odds, we’ve somehow managed, by an almost inconceivable stroke of luck, to alight on the truth about the mathematical domain. But a more reasonable response, it seems to me, would be to acknowledge that our line of questioning has revealed a significant tension in our theory of the world and our place in it, a tension that generates pressure to abandon some part of that theory. What we should conclude, given this tension, is that we have in all likelihood gone wrong somewhere: we’re either mistaken about whether our mathematical beliefs coincide with the facts or mistaken about whether those facts are facts about a domain of mind-independent abstract objects.

This is one way of developing what has become known as the Benacerraf–Field challenge for mathematical platonism. The thought, as Field puts it, is that “if it appears in principle impossible to explain” the coincidence between our beliefs about mathematical entities and the facts about them, “then that tends to *undermine* the belief in mathematical entities, *despite* whatever reason we might have for believing in them” (1989, p. 26). If this is right, then it seems as though a commitment to mathematical platonism brings along with it a commitment to suspending judgment about all matters mathematical. And that means we should either give up our mathematical beliefs or give up platonism.^1^

Notice: the challenge here doesn’t require that we begin in a place of Cartesian doubt—we’re not being asked to justify our mathematical belief-forming methods from scratch, without using those methods. What’s at issue is simply whether we can, *given our own theory of the world*—where this includes all of our mathematical beliefs—explain our ability to get at the mathematical truth. Insofar as this task seems impossible, says Field, our beliefs are undermined. The only epistemological premise needed here, then, is a kind of coherence constraint:

### Field’s Thesis

We can’t justifiably hold on both to our beliefs in a domain and to a theory that makes it impossible in principle to explain those beliefs’ accuracy.

And this looks like a fairly minimal constraint, not a gateway to global skepticism.

The Benacerraf–Field challenge has had a profound impact, both on research in the philosophy of mathematics and on theorizing about abstract objects more generally.^2^ But even this understates its influence. The challenge is, after all, quite widely applicable—a version of it can be advanced against any theory on which there’s no apparent connection between the facts in some domain being what they are and our having the beliefs we do, regardless of whether that domain involves abstracta—and so analogous reliability challenges have been advanced against standard theories in various domains, including both logic and morality.^3^ So it turns out that the challenge, if cogent, has the power to show that our claims to knowledge in a wide variety of domains are simply incompatible with standard theories according to which the facts in those domains are mind-independent.

Unsurprisingly, then, theorists attracted to mind-independence theories in the relevant domains have tried to show that the challenge is *not* cogent. And though these arguments take many forms, I think it’s fair to say that they’re all rooted in misgivings about the notion of explanation. The conditions for adequate explanation are, after all, notoriously difficult to pin down in general, and the present context is no exception: no consensus has ever emerged about just what would count in this context as a satisfactory explanation of our reliability. (Note that the notion of reliability we’re working with here is not to be understood in modal terms—to say that we’re reliable in some domain is just to say that, here in the actual world, most of our beliefs in that domain are true.) And this has led theorists interested in resisting the challenge to suspect that there just isn’t any precisification of the notion of explanation on which the challenge has genuine bite. As Clarke-Doane puts it: “There does not seem to be a sense of ‘explain the reliability’ in which it is plausible *both* that it appears in principle impossible to explain the reliability of our mathematical beliefs [construed platonistically] and that the apparent in principle impossibility of explaining their reliability undermines them” (2016b, p. 36).

What we must show, in order to show that this suspicion is mistaken, is that, *contra* Clarke-Doane, there’s a conception of what an explanation of our reliability would consist in that meets the following constraint:

### Clarke-Doane’s Constraint

An adequate conception of what it would take to explain our reliability must be such that, given that conception, it’s plausible both that (i) standard mind-independence theories of a domain make it impossible in principle for us to explain our reliability in that domain and that (ii) it’s irrational to hold on both to our beliefs in a domain and to a theory that makes explaining our reliability in that domain impossible (i.e., Field’s Thesis is true).

And I want to suggest that there is available a certain modal conception—i.e., one on which explaining our reliability involves giving an account on which the coincidence between our beliefs and the facts is, in a particular sense, modally robust, so that any theory that entails that the coincidence is *not* robust in the relevant sense thereby makes our reliability inexplicable—that does meet this constraint.

Modal conceptions aren’t novel. Field himself, for instance, sometimes suggests that the reason certain theories make our reliability inexplicable is that they entail that our beliefs in the relevant domains aren’t sensitive to the facts—for instance, when he says that the challenge “seems to arise from the thought that we would have had exactly the same mathematical or logical beliefs, even if the mathematical or logical facts were different” (2005, p. 81). But various arguments have been taken to show that such conceptions can’t meet Clarke-Doane’s Constraint.

Some of these arguments do show that particular modal conceptions don’t meet both conjuncts of the constraint. A straightforward sensitivity-based conception of the kind suggested by Field, for instance, turns out not to meet conjunct (ii): if a jury member, in a case where there’s abundant evidence of the defendant’s guilt, believes that there wasn’t an elaborate conspiracy in which “the defendant was framed and all the evidence planted” (White 2010, p. 581), that belief is obviously insensitive, but its insensitivity doesn’t seem to reveal any tension in the jury member’s theory of the world and her place in it.^4^ But as I discuss elsewhere,^5^ well-motivated modifications are available that will result in a sensitivity-based conception that isn’t vulnerable to counterexamples. Hence my own view that what’s needed here is a modified sensitivity-based conception.

In this paper, though, my concern is not with counterexamples to particular conceptions but with a more general objection, one that purports to show that no reasonable modal conception has any hope of meeting conjunct (i) of Clarke-Doane’s Constraint. On the basis of this objection, it has in recent years become standard practice to dismiss modal conceptions altogether, and so my task here is to show that this objection is based on a mistake.

The objection in question is that the necessity of the facts in the domains in question makes it trivial, even given a mind-independence theory, to show that the coincidence between our beliefs and the facts is modally robust in any sense that could reasonably be required—after all, if there are no worlds where the facts differ, then believing what we actually believe guarantees that we aren’t going to go wrong, in which case no reasonable modal conception can deliver the verdict that standard mind-independence theories make our reliability inexplicable.

The problem with this objection—call it *the objection from necessity*—is that it relies on the assumption that the worlds across which modal robustness is to be evaluated, for the purposes of the Benacerraf–Field challenge, are the *metaphysically* possible worlds, and there’s good reason to think this assumption is false. Indeed, I suspect that the only reason this assumption has ever been taken to be at all plausible is that it has never been made fully explicit what the motivation is for accepting a modal conception in the first place: as I show below, when this *is* made explicit, it becomes clear that what motivates such a conception also motivates an understanding of modal robustness in terms not of metaphysically possible worlds but of epistemically possible worlds. So the metaphysical necessity of the facts in the relevant domains is entirely irrelevant—what matters is whether there are *epistemically* possible worlds where those facts don’t obtain. And given a reasonable semantics, there are indeed such worlds. The grounds on which modal conceptions are so often dismissed, then, are simply mistaken: the metaphysical necessity of the facts in the relevant domains is just not relevant to whether such a conception can meet Clarke-Doane’s Constraint, and so the objection from necessity has no tendency whatsoever to suggest that the Benacerraf–Field challenge, interpreted in terms of such a conception, fails to be cogent.

## Understanding the challenge (modally interpreted)

The observation that motivates the Benacerraf–Field challenge is that, on certain standard theories of (say) the mathematical domain, the mathematical facts don’t depend in any way on the natural facts. Likewise, the natural facts don’t depend on the mathematical facts, nor is there any third domain on which both the mathematical facts and natural facts depend. In short, these theories entail that there’s no connection at all between the natural facts being what they are and the mathematical facts being what they are. But on any naturalistically respectable understanding of how our cognitive faculties work, the natural facts fully determine what our attitudes, including our beliefs, are—our attitudes, after all, are instantiated by our brains. These theories of the mathematical domain, then, don’t seem to leave room for any connection between our beliefs being what they are and the mathematical facts being what they are. And analogously for relevantly similar theories of other domains. For convenience, let’s say that, when a theory commits us to this sort of lack of connection, it’s an instance of *orthodox realism*.

Implicit in orthodox realist theories is a certain tension: by accepting such a theory, we commit ourselves to accepting that our beliefs in that domain coincide with the facts while also accepting that those beliefs aren’t connected to those facts in any way that might give us the resources to explain that coincidence. The Benacerraf–Field challenge is designed to exploit this tension. And the way it does so, recall, is by appeal to Field’s Thesis, which states that we can’t justifiably hold on to our beliefs in a given domain while also accepting a theory on which the accuracy of those beliefs is in principle inexplicable. Insofar as orthodox realism really does rule out the possibility of explaining the coincidence between our beliefs and the facts, Field’s Thesis entails that, if we accept orthodox realism about a domain, we must give up our beliefs in that domain. Or, contrapositively: if we’re to avoid being led into skepticism in that domain, we must reject orthodox realism.

The question we must answer is whether there’s any conception of what an explanation of our reliability would consist in on which it’s plausible that Field’s Thesis is true. And modal conceptions—i.e., conceptions on which explaining our reliability would involve giving an account on which the coincidence between our beliefs and the facts is in some sense modally robust—have sometimes been taken to fill the bill. For instance, Field, as mentioned, has suggested that explaining our mathematical reliability would involve giving an account on which each of our beliefs with mathematical content *p* meets the following modal constraint:

### Sensitivity

If it weren’t the case that *p*, we wouldn’t believe that *p*. (Or, equivalently: in (the vast majority of) the nearest worlds where it’s not the case that *p*—even if those worlds are very far from the actual world—we don’t believe that *p*.)^6^

And Warren (2017) has defended a different modal conception.^7^ He suggests that answering the challenge here would involve giving an account on which, roughly, each of our beliefs in a given domain meets both of the following constraints:

### Safety

If it were the case that we believed that *p*, it would be the case that *p*. (Or, equivalently, since the actual world is a world where we believe that *p*: in (the vast majority of) nearby worlds—worlds very similar to the actual world—where we believe that *p*, *p*.)

### Adherence

If it were the case that *p*, we wouldn’t believe it’s not the case that *p*. (Or, equivalently, since the actual world is (we’re supposing) a world where *p*: in (the vast majority of) nearby worlds—worlds very similar to the actual world—where *p*, we don’t believe it’s not the case that *p*.)^8^

Both Safety and Sensitivity are meant to rule out *false positives*—i.e., worlds where we falsely believe that *p*. Safety rules them out in nearby worlds, and Sensitivity rules them out in the nearest worlds where it’s not the case that *p*, regardless of how nearby those worlds are. And Adherence is analogous to Safety but is instead meant to rule out nearby *false negatives*—i.e., worlds where we falsely believe that it’s not true that *p*.^9^ Note also that, to make sense of the claim that the main counterfactual formulations of Safety and Adherence are equivalent to the corresponding parenthetical formulations, we must suppose that the counterfactual formulations are to be understood not in accordance with the usual view, on which counterfactuals with true antecedents are trivially true, but in accordance with Nozick’s suggested alternative, on which, for a counterfactual with a true antecedent *p* to be true, the consequent must be true not only in the actual world but also “in the ‘close’ worlds where *p* is true”—the consequent must remain true “for some distance out in the *p* neighborhood of the actual world” (1981, p. 176).^10^

## The objection from necessity

Suppose some modal conception can indeed meet conjunct (ii) of Clarke-Doane’s Constraint—i.e., can validate Field’s Thesis. What about conjunct (i)? Is it plausible that some such conception can meet conjunct (i) as well—i.e., that, on some some such conception, orthodox realism about a domain *D* really does entail that our reliability in *D* is inexplicable?

It *is* plausible, at least initially. Consider, for instance, Field’s conception, on which explaining our reliability would involve giving an account on which our beliefs satisfy Sensitivity. Given orthodox realism about *D*, there’s no connection between our beliefs in *D* being what they are and the facts being what they are, and so we plausibly would have the same beliefs in this domain irrespective of what the facts were. And if that’s right, then orthodox realism entails that these beliefs fail to satisfy Sensitivity, in which case orthodox realism certainly does make it impossible to explain our reliability, on Field’s conception.^11^

But there’s a problem: as suggested above, the objection from necessity, if successful, shows that no modal conception can meet conjunct (i). The worry can be put as follows. In most of the domains about which orthodox realism has been taken to be attractive—e.g., the mathematical, logical, and moral domains—the facts are necessary, and so there exist no possible worlds where those facts are different. So—given that our (say) mathematical beliefs are true here in the actual world, as we’re supposing they are—it’s trivially easy, even for orthodox realists, to show that those beliefs meet modal conditions such as Sensitivity, Safety, and Adherence. Consider first that, insofar as *p* is necessarily true, Sensitivity is a counterfactual with a necessarily false antecedent—i.e., a counterpossible—and Safety is a counterfactual with a necessarily true consequent. Both of these counterfactuals, then, are, on the standard semantics, trivially true. For recall: a counterfactual with antecedent $\varphi$ and consequent $\psi$ is true just in case either

1. those $\varphi$-worlds most similar to the actual world are also $\psi$-worlds or
2. there are no $\varphi$-worlds.

And the second disjunct guarantees the truth of any counterpossible, while the first guarantees the truth of any counterfactual with a necessarily true consequent. More generally, *any* condition ruling out false positives is trivially satisfied if *p* is necessarily true: if every world is a world where *p*, there can be no worlds where we falsely believe that *p*.^12^

As for Adherence and similar conditions ruling out nearby false negatives: in order for such a condition to be true, it’s sufficient that there be very few nearby worlds where we believe it’s not the case that *p*. But this, as Clarke-Doane (2015, 2016a, 2016b) and Baras (2017) have pointed out, requires only that there be some modally robust explanation for our having the beliefs we do in the relevant domains, so that there aren’t nearby worlds where those beliefs are different. And it seems that there’s going to be some such explanation, presumably an evolutionary one, in the case of our basic mathematical, logical, and moral beliefs.

What are we to do in the face of this objection? Insofar as we want to maintain that conjunct (i) of Clarke-Doane’s Constraint can indeed be met, there are only three possible responses:

- insist that, despite the argument given by Clarke-Doane and Baras, our beliefs in the relevant domains fail to satisfy Adherence (or some other condition ruling out false negatives);
- abandon altogether our modal conception of what it would take to explain our reliability; or
- insist that the counterfactuals under discussion are, in this context, not correctly analyzed via the standard semantics.

Recall, though, that, in order for the Benacerraf–Field challenge to be cogent, our conception must meet conjunct (ii) as well—i.e., must validate Field’s Thesis. Given this requirement, neither (a) nor (b) is a good response here. In the remainder of this paper, I explain why that is, and then I develop and defend response (c).

## Against response (a): the irrelevance of false negatives

Response (a)—i.e., insisting that our beliefs in the relevant domains, on orthodox realism, fail to satisfy some condition ruling out false negatives—has been endorsed, for instance, by Warren (2017, p. 1659): he claims that “selection pressures are simply too coarse-grained for it to be true that we have the same *X*-beliefs [e.g., mathematical or moral beliefs] (even limited to ‘core’ beliefs) in all nearby scenarios”, in which case appeals to evolutionary explanations of our having the beliefs we do can’t help us to secure Adherence. But there’s no need to evaluate this claim. Even if Warren is right, no approach that relies on Adherence (or any similar principle) is tenable: it’s just not plausible that a belief’s failure to satisfy a condition ruling out false negatives is undermining in the way Field’s Thesis requires.

The problem is that a false negative, if the belief in question is our belief that *p*, is simply a world where *p* is true but where we believe the opposite, a world where we falsely *disbelieve* that *p*. And the existence of worlds where we’re mistaken in *disbelieving* that *p* can’t on its own—i.e., unaccompanied by the existence of false positives—give us any reason whatsoever to suspect that, here in the actual world, we’re mistaken in *believing* that *p*. Such worlds, after all, aren’t worlds where we’d have been better off had we been less willing to believe that *p*; they’re worlds where we’d have been better off if had we been *more* willing to believe that *p*. Indeed, it’s plausible that what the existence of such worlds shows, if anything, is that we should be *more* inclined than we currently are to believe that *p*—the way to avoid these false negatives, after all, would be to be more willing to believe that *p*, not less.

An example will help to clarify the point. Suppose that I believe (initially with justification) that parakeets exist but that I then discover that my dispositions are structured in such a way that, in lots of worlds extremely similar to the actual world, I don’t have this belief: I falsely believe that parakeets *don’t* exist. My belief, then, fails spectacularly to satisfy Adherence (along with any other reasonable principle ruling out false negatives). But suppose also that my discovery doesn’t call into question whether my belief satisfies Sensitivity and Safety. Should reflecting on my discovery lead me to abandon the belief that parakeets exist? Certainly not. Consider that, in order for the discovery not to call into question whether my belief satisfies Sensitivity and Safety, it must not call into question my ability to respond correctly when faced with a world where parakeets don’t exist. That is, it must not call into question whether I’m at least responsive enough to the evidence about parakeets to decline, in such a world, to believe that parakeets exist. So what I’ve discovered can only be that I’m disposed to be unduly skeptical about the existence of parakeets.^13^ (The precise nature of these dispositions doesn’t matter very much for our purposes. Perhaps what I learn is that I’m disposed to deny the existence of parakeets unless I have far more evidence of their existence than a reasonable person would need, or perhaps it’s just that I’m disposed to be overly susceptible to the rhetoric of ornithological conspiracy theorists.) If there’s any epistemic problem here, then, it seems to be that I should be far *less* disposed than I am to doubt the existence of parakeets. And it seems quite clear that we shouldn’t take the discovery of facts about my belief-forming dispositions to undermine my justification for a belief if that justification wouldn’t have been undermined had I been *more* cavalier about adopting the belief.

Cases like this one suggest that, given the absence of false positives, false negatives, even those that are very close by, are entirely irrelevant to undermining. And if that’s right, then no conception on which explaining our reliability requires ruling out false negatives has any chance of satisfying Field’s Thesis, in which case we can’t hope to avoid the objection from necessity by appeal to a condition ruling out false negatives. So response (a) turns out not to be a genuine option.

## Against response (b): motivating a modal interpretation

Why not just abandon our modal conception of what it would take to explain our reliability? One reason is that no one has been able to put forward a promising alternative.^14^ A second reason is that, on the assumption that the Modal Security thesis—i.e., the thesis that the only way evidence can undermine our beliefs in a domain is “by giving us reason to doubt that [those beliefs] are both sensitive and safe” (Clarke-Doane 2016b, p. 30)—is true, it’s plausible that, on a conception on which there’s *no* relationship between explaining our reliability and giving an account on which our beliefs meet some modal condition(s), Field’s Thesis is false: if there’s no relationship here, there doesn’t seem to be any mechanism by which learning that our reliability is inexplicable is going to give us reason to doubt our beliefs’ safety or sensitivity. But these are relatively weak reasons. Modal Security is highly controversial, and not particularly well motivated.^15^ And as for the fact that no promising alternative has yet been offered: arguably, this fact gives us some inductive reason to suspect that no promising alternative is forthcoming, but we might have hoped for something more definitive than this.

I suggest that something more definitive can be said here: by reflecting on what would be required in order for for a conception to satisfy Field’s Thesis, we can motivate the claim that explaining our reliability, in the sense relevant to the Benacerraf–Field challenge, would indeed involve showing the coincidence between our beliefs and the facts to be modally robust.

The first thing to note here is that, when we ask for an explanation of our reliability, we’re asking for an account according to which the truth of our beliefs isn’t accidental, isn’t the product of an epistemically problematic sort of luck—this is what’s going to make it plausible that Field’s Thesis is true, that the impossibility of providing an explanation is undermining. And though it’s not immediately obvious just what it would take to rule out the relevant sort of luck, it does seem clear enough that, at the very least, a demonstration would be needed that, given our theory of the domain in question, it was in some sense to be expected that we wouldn’t have mistaken beliefs about that domain.

Explaining our reliability, then, is going to involve giving an account on which it would have been difficult for us to go wrong. And doing this is just going to amount to giving an account on which the coincidence between our beliefs and the facts is modally robust, at least to some degree. For it to be the case that it would have been difficult for this coincidence not to obtain, after all, just *is* for the coincidence not to be modally fragile. We can conclude, then, that, in order to explain our reliability in a domain, it’s necessary to give an account on which our beliefs covary with the facts in that domain across some particular class of worlds.

Of course, we still need to specify what class of worlds is relevant here—i.e., what class of worlds is such that covariance across those worlds is what’s relevant to the nonaccidentality of the truth of a belief. To this end, note that failures of covariance come in two broad sorts, false positives and false negatives, and we’ve already seen that false negatives are irrelevant to undermining. So giving an account on which our belief that *p* is nonaccidentally true will require only ruling out false positives. And that means we need only examine worlds where *p* isn’t true—these, after all, are the only worlds that have a chance of being false positives.

We can further specify our class of worlds by thinking about *which* false positive are such that their existence is relevant to whether the truth of our belief that *p* is accidental. And there are two natural options here. We might take a belief to count as nonaccidentally true as long as there aren’t any (or perhaps are very few) false positives in *nearby* worlds—there’s a case to be made that ruling out false positives in worlds that are similar to the actual world would be enough to show that we couldn’t easily have gone wrong. On this option, what it is for the truth of the belief to be nonaccidental is for the belief to satisfy Safety. Or, alternatively, we might think that covariance across nearby worlds isn’t enough, that, for a belief to be nonaccidentally true, there must be a *connection* between our having that belief and the corresponding fact being what it is. And plausibly, what’s relevant to whether there’s such a connection is whether the belief and the fact move in tandem, at least to some degree—i.e., whether there are false positives in the nearest worlds where *p* isn’t true, regardless of how far from the actual world these worlds are. On this option, what it is for the truth of the belief to be nonaccidental is for the belief to satisfy (something like) Sensitivity. (As I suggested above, this condition will in the end require modification: certain counterexamples show that sensitivity is only an imperfect proxy for the kind of connectedness we’re interested in.) Arguably, the second of these options is the better fit with our above sketch of how the Benacerraf–Field challenge works—the problem with orthodox realist theories, recall, seems to they don’t leave room for any connection between our beliefs and the facts. But we can remain neutral for the purposes of this paper.

Summing up: if our beliefs are to be nonaccidentally true, it must be the case that, across some class of worlds, we don’t mistakenly have those beliefs, where what class of worlds is relevant depends on just what sort of robustness we take to be important. So, in order to give an account on which the truth of our beliefs in some domain isn’t merely accidental, it’s necessary to give an account on which those beliefs meet some modal condition(s): insofar as what’s important is that we avoid nearby false positives, we must give an account on which our beliefs meet Safety, and insofar as what’s important is that our beliefs and the facts move together, we must give an account on which our beliefs meet (something like) Sensitivity.

This, given that explaining our reliability involves giving an account on which the truth of our beliefs is nonaccidental, is enough to motivate the following (partial) modal conception of what it would take to explain our reliability: giving an account on which our beliefs in some domain satisfy the relevant modal conditions, whatever they turn out to be, is necessary for explaining our reliability. Furthermore, it’s highly plausible, on this conception, that Field’s Thesis is true. After all, a theory makes it impossible in principle to meet this necessary condition just in case the theory entails that our beliefs *don’t* satisfy the relevant modal conditions. (If the theory doesn’t entail this, we can coherently supplement that theory with a description of the world according to which our beliefs *do* satisfy the relevant modal conditions, and the result is an account, compatible with the theory, on which our beliefs satisfy those conditions.) And it’s indeed plausible that discovering that our theory entails this would be undermining: our beliefs, insofar as they don’t satisfy those conditions, are accidentally true if true at all, and so, were we to discover that, given our theory of some domain, our beliefs in that domain don’t satisfy those conditions, we plausibly would be required to either give up the beliefs or abandon the theory that put us in this position.

I take it that, given that we can motivate a modal conception in this way, response (b) to the objection from necessity—i.e., abandoning such a conception altogether—is a last resort, to be avoided if possible. So response (c) appears to be all that’s left—we must motivate the claim that, in the context of the Benacerraf–Field challenge, modal conditions such as Sensitivity and Safety aren’t correctly analyzed via the standard semantics. And it turns out that, by keeping in mind that explaining our reliability involves giving an account on which our beliefs are nonaccidentally true, we can indeed motivate this claim.

## Metaphysical impossibility and epistemic possibility

Response (c) is, again, to insist that, standard semantics aside, ruling out false positives is nontrivial even when the contents of our beliefs are necessary truths—i.e., to insist that, even when it’s metaphysically necessary that *p*, so that every possible world is a world where *p*, there are still worlds, metaphysically *impossible* worlds, where *p* isn’t true. To include impossible worlds in our semantics is, after all, to include worlds where the facts in the mathematical, logical, and moral domains are different, and so a semantics that includes impossible worlds is going to be a semantics on which modal conditions ruling out false negatives in those domains aren’t trivially satisfied. Our belief that there are numbers, for instance, won’t turn out to trivially satisfy Sensitivity just because there aren’t any possible worlds where that counterfactual’s antecedent is true (i.e., where there aren’t numbers)—there are, after all, *impossible* worlds where the antecedent is true, and so, for the counterfactual itself to be true, the nearest of these impossible worlds will need to be worlds where we don’t believe that there are numbers.^16^

This strategy isn’t new. Field himself, for example, in the course of discussing the objection from necessity, points out that “even those who think that there is some sort of ‘absolute necessity’ to mathematics may find counter-mathematical conditionals perfectly intelligible in certain contexts”; we seem to be able to say, for example, that “if the axiom of choice were false, the cardinals wouldn’t be linearly ordered, the Banach–Tarski theorem would fail and so forth” (1989, pp. 237–238). And the strategy has received a fair bit of attention in the ensuing literature, with several theorists calling attention to cases in which it’s plausible that we can say nontrivial things about what would be the case were some necessary falsehood true.^17^ Given the plausibility of the examples on offer, we might be tempted to suppose that no further reply to the objection from necessity is needed—if the standard semantics can’t be brought into line with our linguistic intuitions here, we might insist, so much the worse for the standard semantics.

Though I have some sympathy with this position, I think a bit more is owed given that what’s being recommended is so radical a break from orthodoxy. After all, there’s no guarantee that good sense can be made of our linguistic intuitions here—we may just be incoherent. And even if we’re not, even if a semantic system can be constructed that validates these intuitions, it’s a further question whether that system is appropriate for analyzing modal conditions in the context of the Benacerraf–Field challenge. So we should provide a replacement semantics, or at least a sketch of one, on which there are indeed metaphysically impossible worlds where the mathematical (and logical, and moral) facts are different, and we should also provide some reason to think that this replacement semantics is the one we should be working with in a context in which we’re trying to determine whether our own beliefs are merely accidentally true.

It turns out, given some recent work on impossible worlds, that the resources needed to complete the first task are readily available. We need only modify the standard possible world semantics so that its set of worlds includes metaphysically impossible worlds in addition to the metaphysically possible ones, and as Nolan (1997) has made clear, there’s nothing especially mysterious about what sort of modification is needed: impossible worlds, after all, are going to be entities of just the same sort as possible worlds—i.e., state-descriptions, sets of propositions, or something similar—and so we can allow impossible worlds into our semantics simply by relaxing the consistency requirements in virtue of which such worlds were excluded in the first place.^18^ That is, we can provide a semantics that includes impossible worlds simply by treating possible worlds in the usual way and taking impossible worlds to be additional worlds where “*every* formula is treated as atomic” (Priest 2016, p. 2656), so that there are no constraints on an impossible world’s assignment of truth values to formulas.

There are certainly other questions to be answered here, the most significant of which is how similarity between worlds is to be understood in a system that allows impossible worlds as well as possible ones.^19^ But we won’t need to say much about these questions.^20^ What matters, for our purposes, is just that some sensible account is indeed available of how to construct a semantics that includes metaphysically impossible worlds.

Again, though, the fact that there exist formal semantic systems that include impossible worlds doesn’t on its own show that we should rely on such a system in analyzing the modal conditions relevant to the Benacerraf–Field challenge. To show this, we need to motivate the claim that metaphysically impossible worlds are relevant to whether we should take the truth of our own beliefs to be lucky in an epistemically problematic way. This, then, is our final task in this paper.

Note, first of all, that, although it’s indeed plausible that our mathematical, logical, and moral beliefs are necessarily true if true at all, it doesn’t follow that we can be absolutely certain of the truth of those beliefs. Suppose, then, that we aren’t absolutely certain that (say) there are numbers. Then we can say the following: though it’s true that, if there are indeed numbers, there don’t exist any metaphysically possible worlds where there aren’t numbers, there nevertheless do exist *epistemically* possible worlds where there aren’t numbers—there’s a sense of “epistemically possible” on which for there to be epistemically possible worlds where *p* is just for it to be the case that we haven’t definitively ruled out *p*’s being true, so that, from our own perspective, there are situations where *p* that are candidates for being our actual situation.

To be clear, the sort of epistemic possibility that’s in play here is first-personal epistemic possibility of a very liberal sort: all that’s required for there to be epistemically possible worlds where *p*, from the perspective of a given agent, is for the agent to be less than absolutely certain that *p* isn’t the case.^21^ And absolute certainty is an extremely high bar.

Indeed, it’s generally accepted, even by orthodox realists, that, insofar as we’re rational, there’s nothing about which we’re going to be absolutely certain. As Peacocke (2005, p. 747) puts it,

Absolutely indefeasible entitlement is simply not to be had. There can always be some evidence that would rationally make us think we had made a mistake in believing something to be a proof. To have indefeasible grounds we would have to be infallible, and indeed to have conclusive grounds that we are so.

And this applies even to our basic beliefs in domains about which orthodox realism has been taken to be an attractive option: since there can always be new evidence that we’ve made a mistake in applying our methods of belief formation, we can’t rationally be absolute certain of (say) the mathematical fact that there are numbers, or even of the logical fact that everything is self-identical. Insofar as we’re rational, then, the worlds we’ve been describing as impossible—i.e., worlds where the mathematical, logical, or moral facts are different—are epistemically possible in the sense under discussion despite the fact that they are (given the truth of our beliefs in the relevant domains) metaphysically impossible. So, in order to show that we should rely on a system that includes these worlds, we need only motivate the claim that the epistemically possible worlds, not the metaphysically possible ones, are the worlds relevant to Sensitivity and Safety as understood in the context of the Benacerraf–Field challenge.^22^

An initial consideration in favor of this sort of epistemic analysis of our modal conditions is simply that we sometimes do use counterfactuals as tools for reasoning about what our own world is like, and when we do, it’s clear enough that we’re using their antecedents to describe worlds that might turn out to be the actual world—i.e., worlds that are epistemically possible. Consider, for example, a doctor who, when her sick patient begins convulsing, reasons as follows: “If the patient had taken arsenic, he would indeed be convulsing, and this particular symptom is otherwise inexplicable. So I think he did take arsenic”.^23^ It’s quite clear that what’s being described in the antecedent of the doctor’s conditional is a way the actual world might, from the doctor’s perspective, turn out to be—i.e., an epistemically possible world, albeit one that’s only partially described.

A clarification is in order here. I’ve suggested that, insofar as we’re rational, we aren’t going to be absolutely certain of anything. But there’s some room for disagreement about this; perhaps, for instance, introspective certainty of our own experiences and beliefs is available. In order to allow for this, we must, in order for reasoning methods like our doctor’s to be sensible, characterize epistemic possibility even more liberally than suggested above: we must allow that a world can be epistemically possible even if it has indeed been definitively ruled out, as long as it wasn’t ruled out a priori. To see why, consider a (somewhat artificial) variant of our doctor’s case in which she notices that her sick patient is *not* convulsing and reasons as follows: “If the patient had taken arsenic, I’d be having an experience as of the patient convulsing. But I’m having no such experience, so he must not have taken arsenic”. What makes the conditional true here is simply that there are worlds where the patient took arsenic, and the nearest of these are worlds where the doctor is having an experience as of the patient convulsing. But if the doctor can indeed be certain of what she’s experiencing, she can be certain that, in fact, she’s *not* having an experience as of the patient convulsing. So there must be worlds that are epistemically possible in the sense under discussion but that nevertheless are worlds where something is true that the doctor knows by introspection to be false. And something similar goes for our modal conditions, if they’re understood epistemically: in order for it to be a nonvacuous question whether, for instance, we’d believe there to be numbers even if there were no numbers—i.e., whether the nearest epistemically possible worlds where there are no numbers are worlds where we believe that there are numbers—it must be the case that there are epistemically possible worlds where we don’t believe there are numbers. So, again, if we can indeed be certain of what we believe, then there must be worlds that are epistemically possible but that nevertheless are worlds where something is true that we know by introspection to be false. We can make sense of this by noting that, when we use counterfactuals as the doctor is using them, as tools for reasoning about what our own world is like, what we’re doing, more or less, is considering what the world would seem like to us if some particular hypothesis turned out to be true. And if this is what we’re doing, we need to be able to consider worlds that seem different to us than the actual world does.

Moving on: the reasoning we’re engaged in when we work through the Benacerraf–Field challenge does appear to be like our doctor’s reasoning in that it’s reasoning about what our world is like: when we ask about our beliefs’ sensitivity and safety, our concern, plausibly, is whether we’re right to feel secure in our position that we’ve successfully picked out the way thing really are from all the ways things might be. And if that’s right, it gives us some general reason to suppose that, when we think about whether the nearest worlds where there are no numbers are worlds where we believe there are numbers, or about whether there are nearby worlds where we falsely believe that there are numbers, we’re thinking about situations that are candidates for being our actual situation. That is, we’re thinking about epistemically possible worlds.^24^

This is plausible enough, I think, but we can make our motivation for an epistemic analysis more definitive by providing a case that makes it very clear that the worlds relevant to whether we should take the truth of our beliefs to be lucky in an epistemically problematic way are epistemically possible worlds rather than metaphysically possible ones. And such a case can be constructed, though doing so requires a bit of setup.

Imagine that the zipper has just been invented and that ‘Julius’ is a name whose reference we’ve fixed via the following agreement: “Let us use ‘Julius’ to refer to the person who, in the actual world, invented the zipper, if anyone did”.^25^ I hereby stipulate that, whatever the status of the word ‘actual’ as it appears in everyday (or philosophical) English, we aren’t here using it as an indexical: as we’re using it, it refers to our world, to the one actualized possibility, even in the mouths of speakers in possible but nonactual worlds.^26^ And that means that, since the actual inventor of the zipper is Whitcomb L. Judson, the name ‘Julius’—as used in the actual world and also as used in possible but nonactual worlds, including worlds where someone else invented the zipper—refers to Judson: when possible but nonactual speakers fix the reference of ‘Julius’ via the description “the person who, in the actual world, invented the zipper”, the world to which they’re referring is our world, not theirs. Now, suppose that Judson’s status as the inventor of the zipper is somehow modally fragile, perhaps because someone else was working on a very similar design at the same time but died in a freak accident just before the design was finished. Consider, then, my belief that Julius invented the zipper if anyone did.

This belief is, of course, true—Julius is Judson, and Judson invented the zipper. Suppose, though, that we’re wondering whether the truth of this belief is merely accidental. Then our question is whether the belief satisfies whatever modal condition is important, whether it be Sensitivity, Safety, or some other condition ruling out false positives.

The answer, if the worlds that are relevant to the satisfaction of the relevant modal condition are the metaphysically possible worlds, is that my belief is certainly not going to satisfy that condition. After all, there are lots of worlds very similar to the actual world where it’s not the case that Julius invented the zipper if anyone did: the worlds where his rival invented it. And my counterpart in each of these worlds takes himself, wrongly, to be in the actual world: he’s just a person who lives in a world where someone other than Judson invented the zipper, not a person who’s somehow aware of his status as a merely possible counterpart. So he believes, wrongly, that ‘Julius’, in his mouth, refers to whoever invented the zipper in his world. And that means each of these worlds is a false positive, a case in which I believe, wrongly, that Julius invented the zipper if anyone did. So my belief doesn’t satisfy Sensitivity, Safety, or any similar condition.

But now we can ask whether this result should lead me to suspect that the the truth of my belief is lucky in an epistemically problematic way. And I take it to be obvious that it should not, for the simple reason that the mistake my counterparts have made is not a mistake I can ever actually make: their mistake arises just from the fact that they are nonactual, and so, though it’s metaphysically possible for me to make that mistake, it’s not epistemically possible. That is, the actual world can’t turn out to be a world in which I make that mistake. And if there’s no way for the actual world to be a world where I make this mistake, there’s no genuine risk of my making this mistake, in which case the fact that I haven’t made this mistake is no accident. In short, there’s no reason for me to be worried by a possibility that can’t turn out to be actual. But if that’s right, then what we have here—on the assumption that the worlds relevant to the satisfaction of our modal conditions are the metaphysically possible worlds—is an example of a belief that flagrantly fails to meet any of those conditions but that I should nevertheless take to be nonaccidentally true.

If, on the other hand, we assume that the worlds relevant to the satisfaction of our modal conditions are the epistemically possible worlds, it turns out that my belief that Julius invented the zipper if anyone did does indeed satisfy those conditions. Again, there are lots of nearby worlds where it’s not the case that Julius invented the zipper if anyone did: the worlds where his rival invented it. But if such a world turned out to be actual, then the name ‘Julius’, in my mouth, wouldn’t refer to Judson—it would refer to his rival. So, although I’d have a belief that I’d express using the *sentence* “Julius invented the zipper if anyone did”, this would be a true belief about the person who invented the zipper, not a false belief about Judson. So I would not, in this situation, believe that Julius—i.e., Judson—invented the zipper if anyone did. Given our epistemic analysis, then, my current belief that Julius invented the zipper if anyone did satisfies Sensitivity, Safety, and any other reasonable condition ruling out false positives.

What we have, then, is a belief with the following features: it satisfies Sensitivity, Safety, and similar conditions if the worlds relevant to their satisfaction are the epistemically possible ones but doesn’t satisfy those conditions if the relevant worlds are the metaphysically possible ones, and it’s clear that learning these facts shouldn’t lead me to conclude that the belief is merely accidentally true. This strongly suggests, even independently of the objection from necessity, that our modal conditions should be analyzed via a semantics whose worlds are the epistemically possible ones rather than the metaphysically possible ones. The reason it’s supposed to be plausible that our modal conception of what an explanation of our reliability would consist in satisfies Field’s Thesis, after all, is that learning that our beliefs fail to satisfy the relevant modal condition (whatever the precise form that condition turns out to take) is supposed to lead us to conclude that these beliefs, insofar as they’re true at all, are true only by accident. And the case of my belief that Julius invented the zipper if anyone did shows that learning of such failures *shouldn’t* lead us to this conclusion unless the relevant modal condition is analyzed epistemically.

But if it’s right that our modal conditions are to be analyzed epistemically, then the objection from necessity fails to show that our modal conception of what it would take to explain our reliability can’t meet conjunct (i) of Clarke-Doane’s Constraint. After all, though our mathematical, logical, and moral beliefs are, if true at all, true as a matter of metaphysical necessity, it nevertheless isn’t the case that the truth of these beliefs is a matter of epistemic necessity—once again, it’s generally accepted that we can’t be absolutely certain of the truth of these beliefs. And that means there are epistemically possible worlds where these beliefs aren’t true. So, since the worlds relevant to the satisfaction of our modal conditions are the epistemically possible ones, it’s not trivial that these beliefs satisfy those conditions.

## Conclusion

What we’ve shown here is that the objection from necessity has no tendency to suggest that the Benacerraf–Field challenge, modally interpreted, fails to be cogent. Since beliefs whose contents are metaphysically necessary don’t trivially satisfy Sensitivity, Safety, or any similar modal condition ruling out false positives, the necessity of the facts in the mathematical, logical, and moral domains gives us no reason whatsoever to suppose that orthodox realists about those domains can consistently hold that our beliefs in those domains satisfy any such condition. And if that’s right, then, given our conception on which a theory that entails that our beliefs don’t satisfy such a condition thereby entails that our reliability is inexplicable, the objection from necessity doesn’t in any way suggest that we were wrong in thinking that orthodox realist theories of mathematics, logic, and morality make our reliability in those domains inexplicable.

So, despite what’s often thought, the objection from necessity just has no bearing on whether some modal conception of what it would take to explain our reliability can be developed that meets Clarke-Doane’s Constraint. And fact, I take it that such a conception *can* be developed: as I’ve suggested, a modified version of Sensitivity is available that perfectly tracks the sort of connection between our beliefs being what they are and the facts being what they are that’s relevant to whether we should take the truth of our beliefs to be lucky in an epistemically problematic way.^27^ Orthodox realism, then, does indeed entail that our beliefs fail to meet that condition, and this failure is indeed undermining. And if that’s right, then the Benacerraf–Field challenge, modally interpreted, is indeed cogent—it does indeed show that our beliefs in a wide variety domains, given standard mind-independence theories of those domains, aren’t justified.

## Footnotes

1 Benacerraf’s (1973) statement of the challenge presupposes a causal theory of knowledge, but causal theories are nowadays rarely endorsed. Field’s contribution is to show that no particular theory of knowledge is essential to the challenge.

2 See Liggins (2010) for a partial overview of the relevant literature.

3 The challenge for logic comes from Schechter (2010, 2013, 2018b), though he, in the end, thinks this instance of the challenge can be met: he argues that an evolutionary explanation is available of our logical reliability. As for morality, it has often been pointed out—by, e.g., Enoch (2010), Clarke-Doane (2016b), Klenk (2017), and Schechter (2018a)—that evolutionary debunking arguments of the sort advanced by Street (2006) and Joyce (2006), as applied to nonnaturalist versions of moral realism, are best understood as instances of the Benacerraf–Field challenge.

4 For application of cases of this sort to the Benacerraf–Field challenge, see, e.g., Clarke-Doane (2015, 2016a, 2016b) and Bogardus (2016).

5 See my “Saving sensitivity”, currently under review.

6 In response to certain problematic cases, this condition and the ones discussed below are usually relativized to methods of belief formation. It’s not obvious, though, that the relevant cases are problematic in the present context, where what’s important isn’t whether the conditions in question are in fact satisfied but whether the believer’s theory of the world makes available an account on which they’re satisfied. In any case, even if there are motivations for relativizing in this context, they’re largely irrelevant for our purposes here, and so we can ignore this complication for the sake of simplifying our discussion.

7 Though Warren expresses some sympathy with the claim that the modal conditions he’s working with are conditions on explanation, his version of the challenge doesn’t go via explicability at all. He simply claims that, unless the relevant modal conditions are met, it’s unlikely that we’re reliable in the relevant domain. Note also that Warren’s terminology is highly nonstandard—I’ve changed it in order to avoid confusion. The condition I call Safety is roughly equivalent to his “Sensitivity” condition, and the condition I call Adherence is roughly equivalent to his “Safety” condition.

8 To state these latter conditions with full precision, we’d need to say just how similar to our own world a world needs to be in order to be nearby in the relevant sense, and we’d also need to say just how many nearby false positives are allowed. But this level of precision isn’t going to be necessary for our purposes.

9 In principle, Sensitivity could be paired with an analogous condition ruling out false negatives at the *nearest* worlds where *p*, but such a condition would turn out to be equivalent to Adherence: since the actual world is (we’re supposing) a world where *p*, the nearest worlds where *p* just *are* the nearby worlds where *p*.

10 The use of Nozick’s suggested picture is no accident—each of the three conditions here is a version of the condition of the same name that appears in the literature on modal conditions on knowledge, a literature that consists largely of responses to and developments of Nozick’s ideas. Sensitivity and (a stricter version of) Adherence were introduced by Nozick himself (1981, chap. 3), though Sensitivity wasn’t given that name until later, and Safety was introduced by Sosa (1999a, 1999b) as a way of remedying various problems that arise for Sensitivity. But the role these conditions play in this literature—as conditions a true belief must meet in order to count as knowledge—is quite different from their role in our discussion here. (It’s worth noting that some later safety theorists, such as Williamson (2000), Miščević (2007), and Pritchard (2009), don’t follow Sosa’s usage: they describe a belief that *p* as “safe” just in case, roughly, we couldn’t easily have been mistaken about whether *p*—i.e., just in case the belief meets the condition I’m calling Safety *and* the condition I’m calling Adherence. And theorists discussing modal approaches to the Benacerraf–Field challenge—e.g., Clarke-Doane (2015, 2016b) and Schechter (2018b)—tend to follow this later usage. As we’ll see in Sect. 4, though, it’s important, for our purposes, to keep conditions ruling out false positives distinct from those ruling out false negatives.)

11 Whether Warren’s conception can satisfy conjunct (i) is not so clear. For reasons explained below, our beliefs will satisfy Adherence as long as there’s some robust causal explanation or other for our having the beliefs we in fact have. And as for whether orthodox realism entails that our beliefs fail to satisfy Safety, this is going to depend on whether the nearest worlds where a given fact in *D* fails to obtain are nearby. But that’s a bit of a problem: for most domains about which orthodox realism has been taken to be an attractive option, the worlds where the facts in those domains are different are, on the usual way of understanding similarity between worlds, very far away (if they exist at all). Note, though, that it’s generally acknowledged—e.g., by Lewis (1973) himself—that there’s not a single objectively correct measure of similarity between worlds—what measure we use in a given context is determined by the relative importance we attach, in that context, to the different respects in which worlds might be similar or different. And orthodox realism does entail that there are important respects in which the nearest worlds where a given fact in *D* fails to obtain are extremely similar to the actual world: all the natural facts, in such worlds, are exactly the same as they are in the actual world. So, if we can motivate the claim that, in the context of the Benacerraf–Field challenge, natural similarities and differences are particularly important, we can thereby motivate the claim that orthodox realism about *D* entails that my beliefs in *D* don’t satisfy Safety.

12 Something like this argument is often attributed to Lewis (1986), but whether it’s really the argument he’s trying to make is doubtful. For discussion, see, e.g., Hale (1994).

13 Luper (2012), working within the literature on modal conditions on knowledge, uses cases with this structure to argue against Adherence, and Sosa (2002) and Kripke (2011) use some structurally similar cases to argue against Nozick’s stronger version of of the condition.

14 Schechter, for instance, registers his “strong suspicion...that that showing that our beliefs are safe and sensitive is not closely connected to explaining their reliability”, but he admits in the same paragraph that he has no alternative conception to offer: “Unfortunately, the nature of explanation is a vexed topic, and I do not have a general account of explanation to provide” (2018b, p. 333).

15 The primary motivation offered by defenders of the thesis is just that paradigmatic underminers do seem to call into question the modal robustness of the coincidence between our beliefs and the facts—see, e.g., Clarke-Doane (2016b, sec. 2.6) and Clarke-Doane and Baras (2019, sec. 2). (Clarke-Doane and Baras do respond to various objections put forward by, e.g., Woods (2018), Faraci (2019), Korman and Locke (2020), and Berry (2020), but these are primarily defensive maneuvers; not much is said about why we should be attracted to Modal Security in the first place.)

16 As for Safety: our belief will satisfy that condition if *either* the nearest impossible worlds where there aren’t numbers are worlds where we don’t believe that there are numbers *or* the nearest such worlds are far from the actual world. (Either the nearest impossible worlds where there aren’t numbers are nearby or not. If so, then these just *are* the nearby worlds where there aren’t numbers, in which case our belief satisfies Safety as long as, in these worlds, we don’t believe that there are numbers. And if not, there are no nearby worlds where there aren’t numbers, in which case our belief trivially satisfies Safety.) As I suggested above, then, if we adopt a conception based on Safety, we’ll be able to return the verdict that orthodox realism renders our mathematical reliability inexplicable only if we can motivate the claim that certain metaphysically impossible worlds where the mathematical facts are different—namely, those where the natural facts are the same as they are in the actual world—count as similar to the actual world. For more on the status of Safety in a semantics that includes impossible worlds, see Melchior (2020), which appeared while this paper was under review.

17 Liggins, to take just one example, asks us to consider the case of Pierre, a mathematician who devotes his life to proving some mathematical conjecture C and is disappointed when its negation is proved instead—in this case, says Liggins, “The question ‘How would Pierre have felt were he to have proved C?’ seems perfectly sensible, and ‘He would have felt happy, having achieved what he most wanted’ appears a plausible answer to it, even though C is the negation of a mathematical truth, and so ‘Pierre proved C’ is necessarily false (at least, granting the assumption that mathematical matters are non-contingent)” (2010, p. 72).

18 Of course, on Lewisian modal realism, it’s plausible that this modification can’t be carried out at all, since there just can’t exist concrete worlds where necessary falsehoods are true (though see Yagisawa 1988). So we’re presupposing here that merely possible worlds aren’t concrete. But this isn’t much of a problem: almost no one is a Lewisian modal realist, and the reason is that almost no one finds it at all plausible that possible worlds are concrete.

19 Note, though, that it’s not obviously more difficult to answer that question than it is to answer the question of how to understand similarity in a system that includes only possible worlds—even the answer to this latter question, after all, is generally acknowledged to be highly context-sensitive. See fns. 11 and 16 above.

20 For further discussion, see, e.g., Brogaard and Salerno (2013) and Berto et al. (2018) in addition to the Nolan and Priest texts mentioned above.

21 Notice, for instance, that this sort of epistemic possibility isn’t the same as the epistemic possibility that corresponds to the existence of a scenario in epistemic two-dimensional semantics as usually presented (see, e.g., Chalmers 2004). The existence of scenarios as standardly defined isn’t liberal enough: such scenarios are, for instance, constrained by logic, so that, by fiat, there don’t exist any scenarios where the logical facts are different than they are in the actual world. (Chalmers (2011, sec. 10) does discuss more liberal conceptions of epistemic possibility whose corresponding scenarios are less constrained. One such conception—that developed by Collin, according to which a proposition counts as epistemically possible for an agent just in case the agent “has not ruled out [that proposition] a priori” (2018, p. 494)—is roughly the conception we need.)

22 Note, too, that, if we can motivate this claim, we’ll have motivated a kind of epistemic understanding of our modal conditions and will thereby have made clear that Williamson’s recent defense of the view that counterfactuals with impossible antecedents are vacuously true is irrelevant to our discussion: Williamson explicitly notes that his discussion “concerns non-epistemic readings of subjunctive conditionals” (2018, p. 357).

23 This example is adapted from Edgington (2008, p. 6), though I’m not endorsing her particular approach to counterfactuals.

24 Cf. Collin (2018, p. 491), who provides (as far as I know) the literature’s only other explicit defense of an epistemic analysis of anti-luck conditions (though his primary concern is with modal conditions on knowledge rather than with the Benacerraf–Field challenge): what motivates an epistemic analysis, for him, is that “we often find ourselves in situations where propositions that are metaphysically impossible are on the table, for instance if we are trying to work out who our biological parents are, from a group of (epistemically) possible candidates”. Note also that the considerations here help us to see why understanding modal conditions in terms of *conceptually* possible worlds—as suggested by, e.g., Street (2008)—is difficult to motivate despite the fact that, plausibly, it would give us the result we’re looking for: conceptual possibility on its own is no guide to the way things will turn out to be. One of the central lessons of Putnam (1962), after all, is that, insofar as there are conceptual necessities at all, they can turn out not to be true.

25 This is a variant of the descriptive name discussed at length by Evans (1979); I’ve added the phrase “in the actual world” to the name’s reference-fixing description.

26 On Lewisian modal realism, there’s no world that counts in any objective sense as the one actualized possibility—there’s just whatever concrete possible world we happen to inhabit. And if that’s right, my stipulation here fails, since it relies on a false presupposition. But this isn’t a problem: we know already that we’re committed to the claim that merely possible worlds aren’t concrete.

27 Again, see my “Saving sensitivity”, currently under review.

## References

[CR1] Baras D (2017). Our reliability is in principle explainable. Episteme.

[CR2] Benacerraf P (1973). Mathematical truth. Journal of Philosophy.

[CR3] Berry SE (2020). Coincidence avoidance and formulating the access problem. Canadian Journal of Philosophy.

[CR4] Berto F, French R, Priest G, Ripley D (2018). Williamson on counterpossibles. Journal of Philosophical Logic.

[CR5] Bogardus T (2016). Only all naturalists should worry about only one evolutionary debunking argument. Ethics.

[CR6] Brogaard B, Salerno J (2013). Remarks on counterpossibles. Synthese.

[CR7] Chalmers DJ (2004). Epistemic two-dimensional semantics. Philosophical Studies.

[CR8] Chalmers DJ, Egan A, Weatherson B (2011). The nature of epistemic space. Epistemic modality.

[CR9] Clarke-Doane J, Shafer-Landau R (2015). Justification and explanation in mathematics and morality. Oxford studies in metaethics.

[CR10] Clarke-Doane J, Leibowitz UD, Sinclair N (2016). Debunking and dispensability. Explanation in ethics and mathematics: Debunking and dispensability.

[CR11] Clarke-Doane, J. (2016b). What is the Benacerraf Problem? In F. Pataut (Ed.), *Truth, objects, infinity: New perspectives on the philosophy of Paul Benacerraf* (pp. 17–43). Cham, Switzerland: Springer.

[CR12] Clarke-Doane, J., & Baras, D. (2019). Modal Security. *Philosophy and Phenomenological Research*. 10.1111/phpr.12643.

[CR13] Collin JH (2018). Towards an account of epistemic luck for necessary truths. Acta Analytica.

[CR14] Edgington D (2008). Counterfactuals. Proceedings of the Aristotelian Society.

[CR15] Enoch D (2010). The epistemological challenge to metanormative realism: How best to understand it, and how to cope with it. Philosophical Studies.

[CR16] Evans G (1979). Reference and contingency. The Monist.

[CR17] Faraci D (2019). Groundwork for an explanationist account of epistemic coincidence. Philosophers’ Imprint.

[CR18] Field, H. H. (1989). *Realism, mathematics and modality*. Oxford: Basil Blackwell.

[CR19] Field HH, Gendler TS, Hawthorne J (2005). Recent debates about the a priori. Oxford studies in epistemology.

[CR20] Hale, B. (1994). Is platonism epistemologically bankrupt? *Philosophical Review, 103*, 299–325.

[CR21] Joyce, R. (2006). *The evolution of morality*. Cambridge, MA: MIT Press.

[CR22] Klenk M (2017). Old wine in new bottles: Evolutionary debunking arguments and the Benacerraf–Field challenge. Ethical Theory and Moral Practice.

[CR23] Korman DZ, Locke D, Shafer-Landau R (2020). Against minimalist responses to moral debunking arguments. Oxford studies in metaethics.

[CR24] Kripke, S. A. (2011). Nozick on knowledge. In *Philosophical troubles: Collected papers* (Vol. 1, pp. 162–224). Oxford: Oxford University Press.

[CR25] Lewis, D. (1973). *Counterfactuals*. Oxford: Basil Blackwell.

[CR26] Lewis, D. (1986). *On the plurality of worlds*. Oxford: Basil Blackwell.

[CR27] Liggins, D. (2010). Epistemological objections to platonism. *Philosophy Compass*, *5*, 67–77.

[CR28] Luper S, Becker K, Black T (2012). False negatives. The sensitivity principle in epistemology.

[CR29] Melchior, G. (2020). Sensitivity, safety, and impossible worlds. *Philosophical Studies*. 10.1007/s11098-020-01453-8.

[CR30] Miščević N (2007). Armchair luck: Apriority, intellection and epistemic luck. Acta Analytica.

[CR31] Nolan D (1997). Impossible worlds: A modest approach. Notre Dame Journal of Formal Logic.

[CR32] Nozick, R. (1981). *Philosophical explanations*. Cambridge, MA: Harvard University Press.

[CR33] Peacocke, C. (2005). The a priori. In F. Jackson & M. Smith (Eds.), *The Oxford handbook of contemporary philosophy* (pp. 739–763). Oxford: Oxford University Press.

[CR34] Priest G (2016). Thinking the impossible. Philosophical Studies.

[CR35] Pritchard D (2009). Safety-based epistemology: Whither now?. Journal of Philosophical Research.

[CR36] Putnam H (1962). It ain’t necessarily so. Journal of Philosophy.

[CR37] Schechter J (2010). The reliability challenge and the epistemology of logic. Philosophical Perspectives.

[CR38] Schechter J, Gendler TS, Hawthorne J (2013). Could evolution explain our reliability about logic?. Oxford studies in epistemology.

[CR39] Schechter, J. (2018a). Explanatory challenges in metaethics. In T. McPherson & D. Plunkett (Eds.), *The Routledge handbook of metaethics* (pp. 443–459). New York: Routledge.

[CR40] Schechter J (2018). Is there a reliability challenge for logic?. Philosophical Issues.

[CR41] Sosa E (1999). How must knowledge be modally related to what is known?. Philosophical Topics.

[CR42] Sosa E (1999). How to defeat opposition to Moore. Philosophical Perspectives.

[CR43] Sosa E, Moser PK (2002). Tracking, competence, and knowledge. The Oxford handbook of epistemology.

[CR44] Street S (2006). A Darwinian Dilemma for realist theories of value. Philosophical Studies.

[CR45] Street S (2008). Reply to Copp: Naturalism, normativity, and the varieties of realism worth worrying about. Philosophical Issues.

[CR46] Warren J (2017). Epistemology versus non-causal realism. Synthese.

[CR47] White, R. (2010). You just believe that because.... *Philosophical Perspectives, 24*, 573–615.

[CR48] Williamson T (2000). Knowledge and its limits.

[CR49] Williamson T (2018). Counterpossibles. Topoi.

[CR50] Woods J (2018). Mathematics, morality, and self-effacement. Noûs.

[CR51] Yagisawa T (1988). Beyond possible worlds. Philosophical Studies.

